# Book Review

**Published:** 1903-06

**Authors:** 


**A Brief Manual of Prescription Writing.** By M. L. Neff, M.D. Pp. 72. Philadelphia: F. A. Davis Company. 1901.—This is a collection of notes, arranged to enable those ignorant of Latin to write an intelligible and correct Latin prescription. It consists of seventy-two pages of text, and rather more of blank forms for the reader's collection of prescriptions, the text comprising a treatise on prescription writing, together with tables of doses and incompatibles; so that the writer has not given himself too much space. However, his work is very carefully and accurately done, and the inflexions of a remarkable number of medical names are given. When we remember that these are derived, not only from Latin, but also from Greek, mediæval, and modern sources, it is clear the task is not an easy one. How many of us can give correctly the genitive of rhus, statice, cocos, or the gender of æther, carbonas, hedeoma? The author favours the rule of writing terms in the metric system with capitals, and those in apothecaries weight with small letters, to prevent confusion, as in the serious difficulty between grains and grams. A list of Latin numerals should have been included, and a much fuller vocabulary given. Elaterium and elaterinum should be added to the list of easily-confused words, and many authorities would demur to the rule that a hypodermic dose should be half that which is given by the mouth.

**Transactions of the Otological Society of the United Kingdom.** Vol. III. London: J. & A. Churchill. 1902.—In this volume are recorded the proceedings of the Otological Society in its third session, that of 1901–1902, together with a list of the officers and members of the Society, and of its rules and regulations, etc. The Society, though in the words of its President, Professor Urban Pritchard, hardly yet out of the stage of infancy, had at the beginning of this session eighty-two members, and other members have since been added. As only those who have made otology a special study are eligible for membership, we consider the Society has made an excellent start. The most important paper in the present volume is Arthur Cheatle's now well-known "Report of an Examination of the Ears of a Thousand School-children." The otological examination of these children, who were pupils of the Hanwell District School, showed that no less than 520 of 1,000 were considerably deficient in hearing power. Cheatle writes: "It was little less than appalling to realise that 335 out of the 1,000 children examined were, or had been, the subjects of discharge from one or both ears; 88 of these might be spoken of as walking on the edge of a precipice, and 6 as being at least half over." In the discussion on this report, George Murray and Drs. Permewan and Macnaughton Jones contributed statistics of a similar grave import. The Society, on the strength of this, unanimously resolved to urge the central education authorities to take steps to have the ears of all children attending the State schools thoroughly and systematically examined and treated. All the papers reported in the transactions are of importance and interest to otologists.

**The Ambulance in Civil Life on Land and Sea.** By Reginald Harrison. Pp. 39. London: John Bale, Sons & Danielsson, Ltd. 1902.—This interesting pamphlet, which has reached its fifth issue, was originally delivered as a Presidential Address at the Liverpool Medical Society. It originated in journeys the author took to the United States and Canada, where he was much struck with the very effective street-ambulance service in connection with the hospitals, and reflected on the insufficiency of such appliances in this country. He gives an account of the working of the system, which is interesting and instructive, and brings it forcibly before us how much we are in need of such a system here, not only for dealing with street accidents, but for private invalid transport. It is really astonishing how we are satisfied with our existing means of dealing with such cases. A surgeon who sees an acute abdominal case which has to be immediately removed to a hospital, can obtain no effectual means of transport, no stretcher to carry the patient from his house: he must be carried downstairs anyhow, handled with much inconvenience into a cab, and as clumsily handled out again at the other end. The annual cost of such an ambulance, with man and horse, is estimated at £227 a year, but in moderate-sized towns the man and horse could be employed otherwise when not engaged in ambulance work, so that the cost need not be so much. There surely would be demand for its use at a charge among the wealthier class of patient. We are pleased to learn that the St. John's Ambulance Association provide some such system in our own city.

**Official Year-Book of the Scientific and Learned Societies of Great Britain and Ireland.** Nineteenth Annual Issue. London: Charles Griffin and Company. 1902.—It is impossible that a work of this kind shall be year by year correct if the secretaries of the various societies do not give themselves the small trouble of correcting the annual return. The Bristol Literary and Philosophical Club has the name of a deceased secretary, and a "No return," with a star indicating the absence of such return for two years.

**Physician and Friend.** Edited by GEORGE SMITH, C.I.E., LL.D. Pp. 218. London: John Murray. 1902.—This is an autobiography of Alexander Grant, F.R.C.S., who for many years took an active part in the organisation of the Medical Service of India, and who was a personal friend, as well as medical attendant, of the Marquis of Dalhousie. To this latter fact we owe much of the interest of the volume, for we get an excellent outline of the character and work of the famous Governor-General, told in plain but graphic language. The rest of the book is made up of an introductory chapter on the Medical Service of the East India Company and numerous letters from Lord Dalhousie. There are half-a-dozen portraits and illustrations. Dr. George Smith, who was formerly India correspondent of *The Times*, has done his editorial work well, and the book is as readable as such biographies usually are. Grant was brought into contact with many men whose names are associated with the growth and maintenance of our Indian Empire; and he was actively engaged as military surgeon in the first Chinese war, where he became practically acquainted with military surgery. The whole is a record of a useful and honourable life, not of brilliant achievement or of genius, but of steady perseverance and devotion to duty; and is valuable for the sidelights it throws on Indian affairs during an eventful period.

**Regional Anatomy.** By Richard J. H. Berry, M.D. New and Revised Edition. 3 vols. Edinburgh: William Green and Sons. 1902.—This work is published in three volumes. Vol. I. comprises the upper and lower limbs, Vol. II. the abdomen and thorax, and Vol. III. the head and neck. It is intended as a reliable introduction to the science of human anatomy, and is to be completed by two additional volumes dealing with the central nervous system and the joints and special senses. Each volume averages about 200 pages, is devoid of illustrations, and is interleaved with the object of compelling the student to furnish his own drawings. This is an excellent system, and we are glad to hear that this procedure has been proved to work most satisfactorily. The customary guides to the dissector are omitted, but we think this omission a distinct defect. Much care has evidently been bestowed upon the work: it is well printed and well-arranged, and the reputation of its author ensures the accuracy of its contents. We can confidently recommend it to students, and more particularly to those who are able to sketch what they observe, and so make for themselves the most valuable of all anatomical atlases.

**A Handbook of Surface Anatomy and Landmarks.** By Bertram C. A. Windle, F.R.S., M.D. Third Edition. Pp. xiii., 141. London: H. K. Lewis. 1902.—We have already reviewed the second edition of this practical manual,[1] and are glad to see that it is meeting with the success it deserves, especially considering the vast importance of a knowledge of surface anatomy. The most recent surgical methods are naturally apt to escape the notice of the pure anatomist: we may therefore be excused for calling attention to the fact that the lumbar route for colotomy is not now usually employed.

**Outlines of Practical Physiology.** William Stirling. Fourth Edition. London: Chas. Griffin and Co. Ltd.. 1902.—This is still the most complete and best-illustrated text-book on practical physiology, and to this, the fourth edition, there have been made several additions to keep the book up to date. The principal additions have been made to the chapters on the special senses, making the book of greater value to the student of experimental psychology, and the mention of cryoscopy and Mackenzie's clinical polygraph will show how Professor Stirling bears in mind the needs of the medical student. We regret to note several errors that should have been corrected in this edition. Thus the graphic representation of the spectra of HbO and Hb are still transposed as in former editions. We are still told that Schulz recommends the precipitation of primary albumoses by half-saturation with $(NH_4)_2S$. The spectrum and composition of urobilin is said to be "practically identical with choletelin, $C_{16}H_{18}N_2O_3$," and a distinction is still drawn between normal and febrile urobilin. Under the tests for globulin in

[1] *Bristol M.-Chir. J.*, 1897, xv. 192.

urine some mention should have been made that half-saturation with ammonium sulphate brings down primary albumoses also. No qualification of the word peptonuria is made, and no mention of deutero-albumose under this head. We should have welcomed a more full account of the tests in everyday use for the examination of gastric juice, the methods of recognising pentose and other sugars than glucose in the urine, and some description of the reaction of degeneration.

**Aids to Dental Anatomy and Physiology.** By ARTHUR S. UNDERWOOD. Second Edition. London: Baillière, Tindall & Cox. 1902.—The author states in the preface that this edition of his little work "is practically a new book. Not only has almost all of it been re-written and brought up to date, but the scope of the work has been enlarged." In this improved form the book will prove of great assistance and interest to those who wish to acquire "up-to-date" knowledge in the study of dental anatomy and physiology. The dental student especially will find it a most desirable book to read before going up for examination, as it emphasises all those facts he has been trying to master during his curriculum; and the practitioner will appreciate it, as recalling to his memory the results of his own studies and observations ably expressed. The chapter on the nature of the teeth shows one that the author has made a special study of comparative anatomy; that on the development of the teeth, the enamel organ, the dentine organ, and the cement organ fully explains nature's own methods and procedure. The calcification of the dental tissues is most clearly explained, so that it can be readily understood that any flaw in the process of calcification, especially at the junction of the enamel and dentine, is a predisposing cause of caries. The latter part, on practical dental microscopy, is well expressed, and describes clearly the way to make both hard and soft sections of teeth, and enters fully into the methods of staining and setting them up. Having said this much, we recommend the book to the profession for perusal and study.

**Anæsthetics.** By J. BLUMFELD, M.D. Pp. 109. London: Baillière, Tindall & Cox. 1902.—Dr. Blumfeld is to be congratulated upon the clear and thoughtful way in which he has laid before his readers the practical points bearing upon the administration of anæsthetics. By carefully arranging his subject and by tabulating the more important details, as well as by providing an excellent index, the author has made the book efficient for teaching purposes and convenient for reference. There is a statement on page 11 which calls for comment. Chloroform with a specific gravity of 1.5 is recommended, and the addition to it of slaked lime is mentioned as a preservative. Since Professor Ramsay's paper on the subject in the *Lancet* of 23rd January, 1897, many experiments have been conducted by David Brown, F.C.S., F.R.S.E., and his conclusions were published in the *Pharmaceutical Journal* of 24th December, 1898, and seemed to prove beyond a doubt that the presence of slaked lime accelerated decomposition of chloroform, and that the drug with a specific gravity 1.5 deteriorated in five days, whereas chloroform with a specific gravity of 1.49 remained unchanged for many weeks. The reference made by Dr. Blumfeld to carbonyl chloride will, however, suggest to all students the necessity of becoming acquainted with the smell of this product of decomposition, so that its unwelcome presence may readily be detected. Every italicised sentence throughout the book should be carefully noticed, especially the one which occurs upon page 17. On page 33, "amyl nitrate" is, of course, a misprint for "amyl nitrite." We heartily recommend this book as being full of practical information set forth clearly and concisely.

